# Review of the Sensory and Physico-Chemical Properties of Red and White Wheat: Which Makes the Best Whole Grain?

**DOI:** 10.3390/foods9020136

**Published:** 2020-01-28

**Authors:** Sara Grafenauer, Chiara Miglioretto, Vicky Solah, Felicity Curtain

**Affiliations:** 1 Grains & Legumes Nutrition Council, Mount Street, North Sydney 2060, Australia; Felicity.Curtain@outlook.com; 2 School of Medicine, University of Wollongong, Northfields Avenue, Wollongong 2522, Australia; chiara.miglioretto@gmail.com; 3 School of Molecular and Life Sciences, Curtin University, Perth WA6158, Australia; V.Solah@curtin.edu.au

**Keywords:** red wheat, white wheat, whole grain, sensory, taste, wheat, bread, noodles

## Abstract

Establishing sensory and physico-chemical differences between products made from red and white wheat may guide the choice of wheat for use in whole grain and high fibre products. As sensory acceptance is key to consumption, this scoping review aimed to document sensory and physico-chemical research demonstrating quantitative differences in red and white wheat and the associated bran. The following databases were systematically searched following the PRISMA protocol: PubMed, Medline, Scopus, CINHAL and ScienceDirect (1990–2019). Of 16 studies, 13 were sensory studies with 529 participants (six of which included quantitative analysis) and three additional quantitative studies. Overall, 10 studies were in favour of white wheat (seven sensory studies, two focused on quantitative analysis and two with additional quantitative studies). Whole grain (wholemeal) bread, pita bread, crackers, noodles, tortillas, flour, intact grains and bran were examined. Aside from the seed coat colour, levels of bound versus free phenolic compounds and polyphenol oxidase activity appeared most responsible for the differences in red and white wheat. Ensuring the sample size for sensory studies are large enough to detect between-group preferences and linking to physico-chemical analysis are recommended. Attention to blinding techniques in sensory testing and use of food products realistically and consistently prepared with commercial potential are also suggested. This scoping review provides confidence in preference for white wheat for whole grain products, particularly for breads, tortillas and in the choice of white wheat for products suitable for the Asian market.

## 1. Introduction

Although there are thousands of genes in every cell of a wheat plant, just three determine the colour of the kernel or seed coat, such that, if none of the three genes code for red, the wheat bran is white [1]. Potentially, there are variations in bran from white to black, and red to blue [2], but the main commercial varieties are red and white wheat. White wheat is the main type of wheat grown in Australia, a decision taken in the 1920s, whereas other countries, including the U.S. and those of the Black Sea, are more focused on producing red wheat [2]. Red and white wheat differ in their suitability to climates as red wheat is more resistant to sprouting and white wheat is better suited to dry conditions at harvest time [3]. The nutrition composition of red and white wheat (from U.S. data) indicate that the intact grains are near identical, with only minor differences between micronutrients such as iron, zinc, phosphorus and potassium (slightly higher in white wheat), and magnesium, copper and niacin (slightly higher in red wheat) [2]. Regardless of the type of wheat, whole grain wheat is nutritionally superior to refined grain. In Australia, whole grain is defined as the intact grain, or the dehulled, ground, milled, cracked or flaked grain where the constituents—endosperm, germ and bran—are present in such proportions that represent the typical ratio of those fractions occurring in the whole cereal, and includes wholemeal [4]; this is in-line with a new global definition [5]. Comparisons with refined flour point to major differences in fibre and a range of micronutrients such that baking flour, in many countries, is fortified. This includes the U.S. and Australia, where bread making flour is fortified with thiamin, folate and iodine (from iodised salt) [4]. Doblado-Maldonado et al. [2] also presented an analysis of total and extractable phenolics, showing higher total phenolics in red wheat. 

Manufacturing grain products, particularly whole grain products, can be complicated by higher levels of these volatile and potentially bitter phenolic compounds mostly derived from the bran [6]. However, it is thought that white wheat is less bitter than red wheat, and may have broader acceptance [2], particularly among children who tend to be more sensitive to bitter tastes [7]. An advantage for wheat is its versatility within food products, yet for more than 20 years, preference for white wheat over red wheat in grain-based foods has been debated. While some researchers claim that only anecdotal evidence supports white wheat as the preferred option [8], it has been generally acknowledged throughout the industry that a preference exists for white wheat [3] for whole grain bread, breakfast cereal and bakery flour. This is also the case in Asian countries in which noodles, steamed buns and dumplings are produced (from refined flour) where bright, white grain products are desirable [1,9,10]. However, these perspectives have relied on assumptions, and a gap in the literature based on the evidence for preferences has remained.

Globally, diets low in whole grain rank second to diets high in sodium in terms of risk for mortality, and are the leading risk factor for disability adjusted life years (DALYs), leading to 82.5 million DALYs [11]. Although the Australian Dietary Guidelines recommend ‘mostly whole grain’ [12], consumption studies suggest that only about one-third of Australians meet the recommended Daily Target Intake (DTI) of 48 g [13] and intakes are low internationally. Increasing the number of acceptable whole grain products in the food supply may be the first step to increasing consumption of whole grain, and in order to do this, identifying sensory concerns related to whole grain foods is a priority [14]. A number of studies have assessed consumer acceptance of whole grain foods [15,16,17,18,19]; however, consensus regarding the variety of wheat is yet to be determined. Comprehensive studies on the variance in flavour profiles of wheat varieties are not available [14]. Yet, resolving the issue of the most suitable grains may be an important factor in producing a diverse range of whole grain foods, facilitating greater mainstream consumption. Sensory attributes include colour, odour, texture and flavour [14], and testing is important to determine taste preference among consumers. It is understood that children and adults differ in terms of their food choice preferences [7,19] and although the sensory characteristics are important, food choices are complex and learning [20], past behaviour, habit and hedonic factors all play a role [21]. The subjective nature of sensory testing and the production of reliable and reproducible outcomes has been questioned [21]. Physico-chemical analysis may help validate differences between red and white wheat found from sensory tests and may be important to understand the specific issues determining food choice and provides an additional form of evidence and suggest new directions for improving acceptance [20]. The aim of this scoping review was to examine sensory and physico-chemical analytical research testing the differences in red and white wheat and the associated bran, and determine if a preference exists, particularly for whole grain and high fibre products. This is likely of interest to those in the food industry, in particular in baking and noodle manufacture. 

## 2. Methods

A search was carried out in September 2019 in the following databases following the Preferred Reporting Items for Systematic Reviews and Meta-Analyses (PRISMA) protocol: PubMed, Medline, Scopus, CINHAL and ScienceDirect, for the period 1990–2019 for original sensory and quantitative physico-chemical studies regarding red and white wheat and the associated bran. The search terms included: red, white, red wheat, white wheat, in conjunction with sensory, flavour profile, preferences, analysis/test, bread, crackers, noodles, flour, buns. Studies were limited to those published in English, in peer reviewed journals, comparing grain products made from red and white wheat or the bran of red or white wheat. Products used in sensory testing were whole grain or high fibre grain foods. Sensory studies included adults and/or children. Quantitative physico-chemical articles examined whole grains or bran. The references in identified studies were also examined individually to supplement the electronic search. A total of 36 abstracts were identified for review and two authors independently screened, coded and evaluated studies for suitability and methodological quality. Studies were excluded if they did not compare red and white wheat or bran. Studies focused on human subjects or used quantitative testing of physico-chemical parameters, or a combination of these two methodologies. This left a total of 16 eligible studies for inclusion. Due to the heterogeneity of studies within this review, and for ease of assessment, each accepted study was described as being either positive when outcomes were in favour of a particular type of wheat or neutral when the findings were more difficult to determine or results were equivocal. 

## 3. Results

### 3.1. Overview of Included Studies

**Figure 1:**
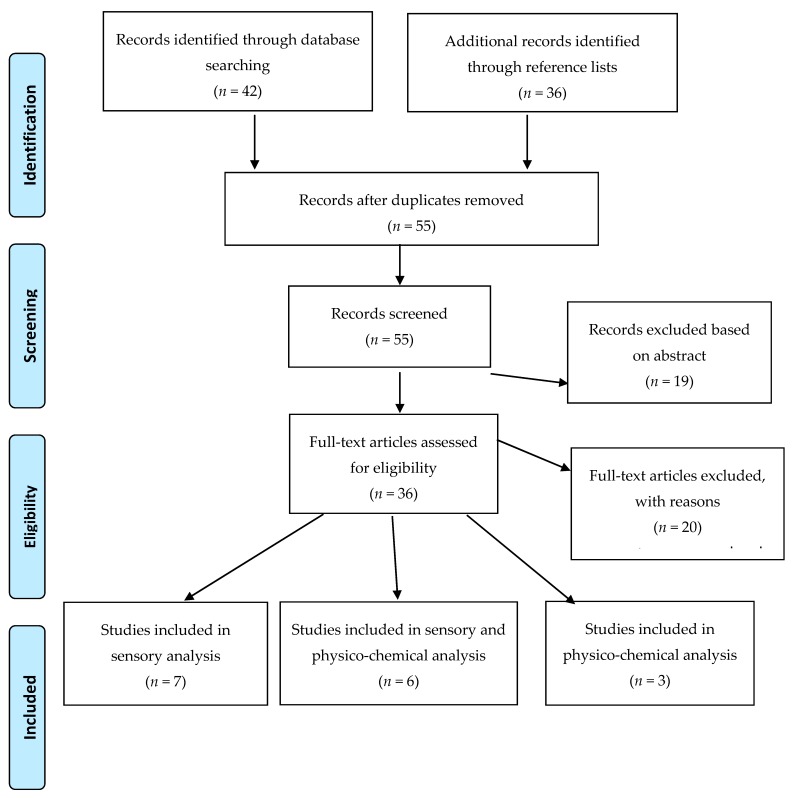
PRISMA diagram.

A total of 16 studies were obtained for this scoping review with the process displayed in the PRISMA diagram (Figure 1). This included 13 sensory studies of 529 participants (range 4–83 participants), six studies also performed physico-chemical analysis (Table 1 and Table 2) and three studies only used physico-chemical analysis (Table 3). Ten of the 16 studies were conducted in the U.S. [1,22,23,24,25,26,27,28,29,30,31,32] with four from Canada [8,33,34,35], one study from Mexico with collaboration from the US [36] and one from China [37]. Studies examined bread (nine studies), crackers (two), tortillas (two), flour (two) and grain (two), and there was one study on each of noodles, muffins, buns, bran and pita bread.

Metrics utilised in sensory testing included overall preference, colour (of crust and crumb; brightness, lightness, greenness, redness, luminance), texture (e.g., hard, firm, tender, gritty), taste, flavour (e.g., bitter, sweet, grainy, mild) and aroma (e.g., sweet dairy, wheaty, bitter, sour, phenolic-like, browned, yeasty). In one study, specific volatile aromatic compounds were identified and assigned an odour profile [28]. Physico-chemical studies focused on phenolic acid content (total, bound and free), polyphenol oxidase (PPO), lightness (L*) and tensile strength. 

Of the seven studies utilising sensory analysis only, five were positive for white wheat [22,23,25,26,33], one study was positive for whole grain (but neutral regarding red and white wheat) [24] and one study was positive for red wheat [8]. There were an additional six sensory studies including quantitative tests, and overall taste preference was clearly positive for white wheat in two studies [29,36], with three neutral studies where the result was more difficult to determine [1,28,34], and one study positive for red wheat [27]. Three included studies used only quantitative analysis and all indicated positive outcomes for white wheat [30,35,37], confirming theories regarding phenolic compounds and PPO which cause browning. These studies were conducted on intact grain, flour and bran. Fourteen studies utilised the whole grain as either intact grain or milled to achieve wholemeal, thus meeting the definition for whole grain, and two utilised only the bran from red or white wheat.

### 3.2. Sensory Studies Evaluating Red and White Wheat Products

Of the 13 included sensory studies, six utilised trained participants, six involved untrained participants and one used a combination of trained and untrained participants. Eight studies applied blinding in the form of red light, coloured samples or by blind-folding participants. Four studies did not use blinding and one study was not clear as to this aspect. While most studies included adult participants, two examined the preferences of children 3–5 years of age; however, these are reported as abstracts.

The earliest study by McGuire and O’Palka [22] examined crust and crumb differences with a small number of panellists who were known to be able to detect flavours. In several tests (over a two-year period) they compared red and white wheat in bread made from wholemeal and refined flour. Colour differences were obvious between the red and white wheat bread in this study and in regards to the wholemeal bread, participants reported that there was bitterness in the red wheat crust and sweetness associated with the white wheat product. Not all the participants detected the difference, though they discerned greater differences between red and white wheat in the crust of the bread compared with the crumb. When a 50:50 mix of flour was used in wholemeal bread with red and white wheat, panellists were not able to note the difference. While results are presented for this study, there was no accompanying discussion. 

The study by Zhang et al. [23] was included in this scoping review although the focus was on the addition of three different particle sizes of bran, which included a comparison between red and white wheat. Bran content and particle size is important, potentially influencing baking performance and loaf quality. Zhang et al. [23] found that there was an overall preference for bread made from soft white wheat bran (10% addition) from a colour, flavour and mouthfeel perspective in comparison to red wheat. Fine bran was also preferred over more coarse milled bran yet the medium bran particle size resulted in the best loaf volume.

Camire et al. [24] made muffins with wholemeal red wheat, white wheat and refined flour. As the red wheat muffins were significantly darker, redder and less yellow than other muffins, dye was used for the refined grain muffins in the sensory tests [24]. Participants (*n* = 66) reported a preference for the red wheat over white wheat muffins (6.9 ± 1.4 versus 6.6 ± 1.3), but there was no significant difference in scores. Perceived healthiness ratings were significantly different between samples; whole red wheat scored highest, followed by whole white wheat and the lowest score given to the refined all-purpose flour muffin. Following provision of health information, there was increased liking for both of the whole grain varieties over refined flour products *(p < 0.05).* The authors suggested that content information may be needed for consumers to help validate choices for whole grain products as the colour was not the only factor affecting preference.

Challacombe et al. [8] reported sensory differences in whole grain products made from red and white wheat (with fine or course bran particle sizes) and product moisture content was in favour of red wheat. Trained panellists (*n* = 13 and 10; bread and crackers respectively) determined the sensory attributes for each of the bread crust, crumb and crackers. They found no differences in bread crust, yet white wheat bread crumb was deemed more bitter and less sweet, while white wheat crackers were harder. White wheat bread (crumb and crust) was deemed more ‘grainlike’ *(p < 0.05)*. A total of 149 untrained participants (73 and 76) were also utilised across the two product types with the same preference for red wheat bread and crackers, although 38% were found to prefer refined flour products [8]. 

Watts et al. [33] utilised Canadian red and white wheat to compare pan (loaf) and pita bread among 10 participants including wholemeal at 95% extraction. Sweeter and milder flavours and lighter colour *(p < 0.05)* were found to be characteristic of white wheat products, whereas red wheat was more bitter and had sour aftertastes. Darker crumb and crusts were also common to the red wheat products and were linked with the higher wheaty flavour intensity, deemed a negative characteristic in this study.

Two abstracts were included in this scoping review, the first published by Keeney et al. [25] examining preferences for red and white wheat bread products among children (3–5 years of age; *n* = 26) and their parents. The sample were already consuming whole grain bread at home; 69% of parents and 72% of children reported consuming whole grain more often than white refined bread. In separate testing, the researchers found that 92% of children preferred white wheat bread, while 73% preferred the red wheat bread with no significant difference in hedonic testing. However, the children consumed significantly more (3.7 g versus 3.3 g) white wheat bread. The second, by Worden et al. [26], examined red and white wheat bread and tortillas among 63 children. Similarly, to Keeney et al. [25], more than half of the parents and children reported consuming whole grain bread (65%), but only 10% of children and 18% of adults consumed whole grain tortillas. Children reported a preference for white wheat bread and tortillas and consumed more of these products but the difference in consumption did not reach significance. The number of parents nor the *p*-values were reported in these abstracts.

### 3.3. Sensory and Quantitative Physico-Chemical Analysis of Red and White Wheat Products

Of the six studies combining sensory and physico-chemical analysis, three utilised very small numbers of participants (*n* < 15), and although they were trained, the results in two did not point to a clear outcome [28,34], and the third was complicated by potential contamination of the sample [27]. The two larger and more recent studies indicated a preference for white wheat, whereas the study by Lang and Walker [1], comparing wheat types in hamburger buns including whole grain, detected a significant taste difference (*p < 0.05*), but participants (*n* = 82) did not prefer one bun type over the other (40% preferred white wheat; 36% preferred red wheat and 24% had equal preference). The white wheat bun contained 7.5 g dietary fibre in the 50 g serving, this is very high in dietary fibre compared to commercially available products and this could have affected findings in this study. Descriptive sensory analysis for flavour difference was not captured.

As indicated, Chang and Chambers [27] utilised six highly trained participants to discern flavour profile differences in bread made of red and white wheat (white and wholemeal). They concluded that the red wheat bread had a more astringent crust but the crumb was sweeter and more dairy-like, while the white wheat bread had a crust that was more toasted and the crumb had a more ‘phenolic-like flavour character’. There were some questions as to contamination of one of the white wheat product samples with machine oil in this study which would have affected the results. In any case, these researchers identified that the bran component of each wheat type was responsible for differences in the breads examined [27]. Just three years later, the same lead author Chang et al. [28] examined the volatile flavour and odour components in bread made from red and white wheat and found 11 of 74 compounds had bread-like odours. Although there were differences between the refined flour and whole wheat breads (15 compounds were significantly higher in whole wheat breads), there were fewer differences between the red and white wheat products using just four highly trained panellists. Ethyl acetate (fruity), ethanol (alcohol), 2-ethyl-3-methylpyrazine (toasted, nutty, brown, burnt peanut, crust-like) and ethyl octanoate (fruity, sweet) were higher in relative quantities in red wheat breads, and 2-butoxy-ethanol (sweet, ether-like *odour*) and 2-furfural (brown) were more abundant in white wheat breads *(p < 0.05*) [28]. The authors comment that the brown, toasted flavours were likely to be the most distinguishing differences (creating bitter flavour) and these were probably due to non-volatile compounds not examined in this study. The very small number of highly trained participants (*n* = 6 and *n* = 4) in both Chang et al. [27] and Chang et al. [28] may limit the generalisability of the results. 

Ramirez-Wong et al. [36] utilised red and white wheat at three different levels of extraction up to a 100% whole grain tortilla that could be scaled commercially. The white wheat flour was determined as the strongest and at 80% extraction had the best colour (L value). The sensory panel accepted tortillas at all levels of extraction but the products were not compared, weakening the analysis. The white wheat product was more flexible and softer at 80% and to a lesser extent at 100% extraction, perhaps due to the higher protein content and gluten. From a production point of view, the white wheat dough was more extensible and had a higher water absorption. It was commented that the higher protein and fibre content of the white wheat tortilla would be of greater nutritional value especially as the 80% and even the 100% extraction product were acceptable via a commercial process. The additional cost to achieve spot-free tortilla was discussed, and the authors point out that some local customs permit darker, spotted product, and hence, use of red wheat in the product would be permissible [36]. However, the efficient extraction from white wheat from 80–100% may point to white wheat as a more economical ingredient as yield is higher and it was the most suitable for whole grain tortillas. 

Challacombe et al. [34] investigated both whole grain bread and crackers made from red and white wheat (at two particle sizes) with a panel of 13 (for bread) and 10 participants (for crackers). Comparisons of red and white wheat total phenolic acid content and phenolic acids were performed and quantified by high performance liquid chromatography. Whole grain bread was reported to have more ‘grain-like, wheat and malted flavours’, whereas the whole grain crackers in comparison to the refined cracker had higher ‘wheat, toasted and earthy notes’. There was a significant difference between red and white wheat in terms of bound phenolic acids with higher levels in white wheat *(p > 0.05)*, but there was no difference in total phenolic acid compounds. In this study, a partial least squares regression was used and demonstrated that total phenolic acids were clustered together alongside the whole grain sensory attributes, whereas for bread crumb both free and bound phenolic acids they were predictive. In crackers, only bound phenolic acids were predictive—hence, moisture may play a role in determining flavour [34]. Phenolic acid profiles, particularly higher bound phenolic acids appear to correlate with favourable sensory characteristics, in this case pointing to a preference for white wheat. Future research is suggested by Challacombe et al. to understand this finding, but there are indications that bound phenolics are not accessible to taste receptors in the oral cavity, and this may provide the difference in sensory studies [14].

A study using near-isogenic lines (related lines), by Talbert et al. [29] tested red and white wheat varieties and PPO activity in bread and noodles. Over two days of testing across 24 panellists, the white wheat bread was found to be consistently and significantly sweeter (*p < 0.01*) on both experimental days and less grainy (*p < 0.01* on day 2). Noodle colour was assessed at time zero and at 24 h after production. Colour brightness (L*) was influenced by white versus red seed colour and by PPO level; however, these findings were inconsistent, yet both varieties of white wheat with high PPO outperformed red wheat at 24 h *(p < 0.001).* The authors suggested that hard white wheat (low PPO) offered the greatest potential end-use quality advantages for Asian noodle performing better in terms of brightness than others at time 0 *(p < 0.05)* and at 24 h *(p < 0.001).* This variety was also suggested for whole wheat bread production, particularly as sweeter-tasting whole wheat bread could help consumers with taste acceptance, a conclusion drawn by others.

### 3.4. Quantitative Physico-Chemical Studies Evaluating Differences in Red and White Wheat

All three physico-chemical studies support a preference for white wheat. The earliest study by Park et al. [30] investigated PPO activity in red (10 varieties) and white wheat (40 varieties) as grain and flour as this enzyme is responsible for browning in finished wheat products, and is often discussed in relation to noodle dough. Specifically, they measured *L* value indicating brightness or a whiter sample. While there was no genotypic or location effects on red wheat in PPO, there was variability in PPO due to growing location affecting white wheat varieties. The authors suggested that for white wheat, selection strategies may be able to be used to help control expression of this enzyme at high levels, through controlling the growing environment. This deserves further investigation as a specific benefit of cultivating white wheat varieties.

Although the aim documented in the article by Kim et al. [35] did not mention a comparison between red and white wheat, they found the phenolic compounds were significantly higher in red wheat bran compared with white wheat bran. These were predominantly in bound form, which were 2.5–5.4-fold higher than free phenolic acids. The range of free phenolic acids extracted from red wheat bran were greater in number and amount (µg/g) compared with the white wheat bran. Acidic and alkaline hydrolysis were used to separate the specific phenolic acids and those separated using alkaline hydrolysis, in particular ferulic acid, had the highest antioxidant activities, whereas trans-cinnamic and vanillic acids were the weakest. Significance was not specifically reported between red and white wheat bran samples for any measure in this study. 

The most recent study was published in 2016 by Ma et al. [37], who compared purple, red and white wheat and found that bound phenolic compounds and antioxidants were highest in purple wheat and soluble phenolics were higher in both purple and red wheat compared to white wheat. However, in this study, white wheat was higher in phenolic acid compounds and biosynthesis gene expression at an earlier stage of development whereas at a later stage, purple wheat was higher [37]. The authors suggest further studies are required to uncover the functions of genes involved in the biosynthesis of different phenolic acid compounds. 

## 4. Discussion

Overall, 10 of 16 studies were in favour of white wheat [22,23,25,26,29,30,33,35,36,37], three were neutral or difficult to determine [1,28,34], and three were in favour of red wheat [8,24,27]. In the studies considered neutral, there were small numbers of participants [28] and in Lang and Walker [1], the white wheat bun was very high in dietary fibre (7.5 g in a 50 g serving), whereas buns on the Australian market currently contain 2.2–12.4 g/100 g and a mean of 4.7 g/100 g (internal GLNC data October 2019). This potentially influenced the outcome among an untrained group of participants. Although the study by Challacombe et al. [34] attempted to discern groups using clusters for overall consumer acceptability, more than one-third of participants preferred the refined grain products and significance was not reported. In the studies supporting red wheat, Camire et al. [24] found no significant difference in ratings although red wheat scored higher than white wheat and the difference was lessoned following the provision of health information. Chang et al. [27] revealed contamination of the white wheat with machine oil, potentially limiting the validity of the outcomes, and Challacombe et al. [8] used products that were not suitable for commercialisation yet these were tested on untrained consumers. 

All but six studies examining whole grain or high fibre food made of white wheat were confirmed to be well suited to bread, pita bread, tortillas and flour. Others also provide support for Asian noodle products [29]. Resolving the dilemma between red and white wheat and highlighting potential areas for future research supporting grain foods may help point to better raw ingredients for healthier whole grain and high fibre products. Together with the Global Burden of Disease data [38], observational evidence consistently supports whole grains as a positive dietary component, exhibiting protective effects against all-cause mortality, cardiovascular disease, type 2 diabetes mellitus, colorectal cancer and weight gain [39,40]. Consumption of whole grain foods using Australian data was calculated from the National Nutrition and Physical Activity Survey (NNPAS) in 2011-2012 at 21 g/day for adults (19–85 years) and 17 g/day for children (2–18 years) [13] and a similar level was derived from the most recent consumption research commissioned by the Grains & Legumes Nutrition Council (GLNC) (*n* = 1121) at 26 g/day (9–70 years) and 16 g/day for children [41]. These levels of consumption are similar to those gathered from the UK [42], U.S.A. [43], Ireland [44] and Singapore [45], while other countries, such as France [46], Italy [47] and Malaysia [48], have far lower intakes. Increased consumption has been achieved in Denmark in recent years from 33 g/day (in 2000–2004) to 55 g/day (in 2011–2014) [49], but as rye bread is a traditional food option [50], this may have been less of a challenge compared with countries where white bread and white rice are staple foods such as in Australia and the Asian region. We would anticipate limited acceptance of dark, heavy rye bread for daily consumption in the Asia-Pacific region; hence, the exploration of wheat varieties which produce acceptable whole grain products, particularly wholemeal bread. 

While a number of studies had assessed general acceptability of whole grain foods [15,16,17,18] including muffins, bread, rolls, breakfast cereals, oats, muesli, pasta, brown rice, cookies, granola bars, cereal bars and other snacks, the potential distinction between red and white wheat in whole grain or high fibre foods for use in these choices had not previously been confirmed. Aside from the obvious genetic colour differences between the red and white wheat seed coat and bran, from a milling perspective there are other advantages to white wheat. It is well established that higher extraction is possible with white wheat when milled to similar colour standards, which is of potential economic benefit [36]; at higher extraction, white wheat remains much lighter in colour [1,36]. This has advantages from a nutrition point-of-view, because higher extraction also permits higher protein and the good quality gluten may be able to overcome the higher proportion of fibre for use in particular whole grain products where fibre interferes with gluten strands, such as in bread [36]. The colour of the bran from white wheat is also more desirable for both refined and high fibre products, as lighter bran is less likely to add dark specks to dough [22,29,33]. When considering grain foods, consumers apparently tend to believe that darker brown breads are healthier, although this may not be true [16], and this feature does not necessarily encourage consumption as a darker crust was considered a negative feature in a number of studies [1,22,33]. It does however point to an additional element influencing sensory acceptability of whole grain foods, concerning the presentation and colour of the product in sensory testing. Smith et al. [7] suggested reducing the visible differences in whole grain bread for sensory testing as this may be key in determining the preferred grain for each product application. However, while this serves as a model in an experimental setting, white wheat is known to produce a lighter colour product and this is likely an additional factor driving preference among consumers [19] and provides an argument for allowing panellists to see the product they are testing. Camire et al. [24] used coloured muffins and consumers possibly found it more difficult to select a preferred option.

Research points to differences in the phenolic profile of wheat including free, conjugated and bound phenolic compounds, with the majority in the bound form in both red and white wheat [51]. While milling removes phenolic compounds from refined flour, by removing the bran, whole grain flour has higher levels of these compounds, potentially leading to the bitter taste and astringency [7,34]. White wheat is reportedly higher in bound phenolics and this may contribute to its perceived sweeter taste, as only free phenolic compounds are flavour-active, adhering to taste receptors [14]. Bound phenolics survive digestion in the upper digestive tract, attaching to the cell wall, and reach the colon where they are released by intestinal microflora [51]. Several authors confirmed differences between red and white wheat [34,35,37], with bound phenolics higher in white wheat and free phenolics up to 2.5–5.4 times higher than bound phenolics in red wheat [35]. Individual phenolic acid concentration was also higher in red wheat bran compared to white wheat bran [35]. In comparison, the total phenolic acids found in oats, corn and rice were less than half that of wheat and rye [50]. Finely milled flour, and the smaller the bran particle size, increased the bioaccessiblity of the free and conjugated phenolic compounds during digestion [6], playing a role in the health of the bowel. Baking, and the chemical changes that occur as a result of heat and the Maillard reaction, may also encourage bitter notes in bread, particularly in the crust [14], but also in dry products such as crackers [34]. Environmental factors also impact the phenolic acid content, and this might explain why white wheat was perceived as more bitter than red wheat in the study by Challacombe et al. [8]. However, this study stands out as an anomaly; other researchers have found red wheat and products made from red wheat were more likely to deliver bitter compounds [23,25,26,29,33].

The PPO content of red versus white wheat may also be a factor driving preference for particular wheat varieties in certain products. The enzyme is not only in wheat, but in many fruit and vegetables such as bananas, apples, potatoes and mushrooms [30] and can cause browning in finished grain products, and therefore, result in poor consumer acceptability. White wheat varied in PPO based on the growing environment and stressors, and therefore, may be more amenable to breeding and selection strategies [30]. In recent years, wheat breeding lines with nil PPO activity have been made available [9]. Still, browning remains a particular issue for noodle dough where brighter, whiter colour is preferred, particularly in Asia [31], and although slightly yellow dough is accepted, red, brown or dull noodles are rejected [9]. Resolving this preference for whiter, brighter noodle dough is considered the ‘holy grail’ of noodle manufacture [9]. In recent research, Australian white wheat, at higher levels of extraction, have been shown to outperform Chinese white wheat for noodle making and steamed buns [10]. Thus, Australian white wheat may have additional benefits to promote the particular features and benefits in export markets with specific breads and for noodle manufacture [52]. Seib et al. [31] also noted that a higher extraction rate was possible for white wheat (65% for white vs. 60% for red wheats) while maintaining a lighter colour noodle and while a low PPO level helps limit browning, swelling and tensile strength are also important features in the manufacture of noodles. Rayas-Duarte et al. [32] also examined red and white wheat in noodles, and in addition to the lighter colour of raw dough, found higher peak viscosity, higher elasticity and better ‘resilience’ in the white wheat samples. Resilience suggests that the noodles have ‘more of a rubbery structure and recover their energy sooner’ and this is considered a valuable attribute in noodles [32].

Recent reviews of the sensory [14] and the bioactive characteristics [2,6] of whole grain and cereal grain foods have been published but do not make a specific assessment of red versus white wheat in whole grain products. Heiniö et al. [14] described a range of product specific sensory challenges for bread, biscuits and pasta and concluded that white wheat provides a more pleasing product, lighter in colour with a milder flavour and together with fine milling, suggesting this as the best option for whole grain products confirming the findings presented here. In contrast, Călinoiu et al. [6] did not examine the difference in types of wheat, just types of grains, pointing to differences between the grain and the bran of wheat, oat and barley. They commented that the phenolic acids alongside the fibre may be the components most responsible for the health effects of whole grains, via the antioxidant and anti-inflammatory effects that these have within the gastrointestinal tract. They acknowledge the importance of the sensory attributes of whole grain products with a lens focused on the bioaccessibility of the bioactive components and nutrients [6]. Yet, these health promoting compounds are also likely to be those that are most problematic in creating acceptable whole grain products. Future research should investigate encapsulation of phenolic compounds, protecting them during food processing, enhancing absorption and improving bioavilability [6], particularly for use in potential functional grain-based foods. The review by Doblado-Maldonado et al. [2] focused on milling and storage of whole wheat flour and acknowledge that there is no standard method for milling whole grain. They indicated limited nutritional differences between red and white wheat and presented data for a range of macro- and micronutrients, and suggested that although red wheat may be entirely suitable as a refined flour (because the bran is removed), it appears to be less suitable for whole wheat baking. The authors stated that white wheat is preferred by consumers [2]. 

A study not included in this review utilised an electronic nose to detect differences in red and white wheat made into bread [53]. They reported that the e-nose was able to detect difference between the crust and crumb of each bread type tested and the red wheat product was clearly discriminated from the white wheat samples and the refined product. They concluded that the bran type manifests volatile characteristics of the genotype and as the white wheat variants were misclassified as refined bread, this indicated that white wheat was milder in aroma compared with the sample containing red bran [54], and is therefore supportive of white wheat. 

There is significant impetus to increase whole grain intakes as a proportion of total grain food consumption for health reasons. Interestingly, Food Standards Australia New Zealand (FSANZ) do not regulate claims describing the amount of whole grain in foods; rather, they just provide a definition for whole grain [55]. In order to provide a framework and guidance to industry and consumers alike, GLNC led the development of the Code of Practice for Whole Grain Ingredient Content Claims (The Code) in 2013. The Code provides guidance on the use of whole grain ingredient content claims outlining cut-off values of at least 8 g per manufacturer serve (contains whole grain), at least 16 g per serve (high in whole grain) and at least 24 g per serve (very high in whole grain) [56] and complements existing food standards. The whole grain DTI was determined by an expert round table [57,58] and is based on the scientific evidence that people who eat at least 48 g of whole grain each day are less likely to develop coronary heart disease [59] and this is consistent with the target used in the U.S. [60]. GLNC also registers whole grain food products that meet the minimum of ≥8g/manufacturer serve. With close to 747 products registered (October, 2019), 24% contain whole grain, 20% are high in whole grain and 56% are very high in whole grain (mostly from the bread and breakfast cereal category), proving that acceptable whole grain foods can be successfully manufactured and for the consumer, achieving adequate whole grain intake within a day is very possible across a small range of foods. According to Mintel, globally there were 44,832 products claiming whole grain content launched from July 2013 to October 2019, and in Australia, 1410 products in the same period (Mintel Global New Product Database, 2019, via subscription service). Growth in products claiming whole grain content has increased 72% (2013–2019) and will likely increase further as regulation in the area is strengthened. 

## 5. Limitations

There were limitations in the studies included in this review, beginning with the raw ingredients through to the food samples used in the sensory tests. Consistency across raw ingredients and product samples for use in sensory analysis are of critical importance and there were issues reported within the studies presented here (e.g., contamination) that affected results. There was an indication that grain varieties are influenced by the environment or growing location and this may be important for specific end product features. This is already a strategy used in Australia for Udon noodles to markets such as Japan [52] and among other grains such as oats, where certain varieties are chosen to support high β-glucan health claims for heart health and cholesterol reduction [2]. 

Several studies utilised products that had been produced in the laboratory, and may not resemble products that would be produced commercially and for this reason, using commercially available product to test consumer acceptance would be recommended [8]. Additionally, inconsistent approaches to production of the whole grain products used in the sensory tests may also have influenced the level of acceptance; for example, in this review, bread baked for a longer time had stronger flavour development than bread baked for a shorter time due to browning of the crust [28]. Furthermore, it has been suggested that adding whole grains in a way that they are not noticed visually or texturally would be the best option for enhancing acceptance among children [19] and there are such products available on the Australian market made from white wheat. 

From a methodological point of view, future sensory studies should ensure panel participants are sufficient in number to allow a preference to be calculated between groups. Consequently, some research presented in this review were not powered to calculate statistical significance or did not present this data. This is problematic considering the nature of sensory testing. By simply asking a participant if they like the food, or which do they prefer, there is greater attention paid than if they were to just eat the food under normal circumstances. Köster suggests that people detect ‘nuances of taste that they had never noticed’ influencing responses even regarding familiar foods and suggest that observation of actual choices and subsequent eating behaviour may be a better [21]. Finally, the included studies were heterogeneous in nature, covering a range of food products with diverse formulations, utilising both highly trained and completely untrained participants and children. These differences also need to be carefully interpreted, and are an important consideration in future research in this area. The studies of children were in abstract form only, limiting the detail reported. 

## 6. Conclusions

The focus of this review was on resolving differences between red and white wheat for whole grain and high fibre foods. Overall, there was support for white wheat for whole grain, high fibre options and in specific grain foods, for example those desired in Asian markets (which are refined). Promotion of Australian grain locally and in export markets should leverage the distinct attributes of white wheat, particularly in light of global growth in the whole grain market. This review provides confidence in preference of white wheat particularly for whole grain bread, tortillas and flour useful for food industry and in Australian export markets. It has been reported that bran colour is not often considered when milling refined wheat flour, but it has a substantial impact on whole wheat flour with significant variation in colour of end products, which may influence consumer acceptance as noted in a number of studies presented. Levels of bound versus free phenolic compounds and PPO activity appear responsible for the differences in red and white wheat in terms of taste and colour (due to browning). With extraction of white wheat of up to 80% found to produce acceptable bright, light coloured whole grain products, there may be sensible economic reasons for selecting this type of grain. The benefits of utilising the whole grain are beyond basic nutrition, with correlations between phenolic compounds, dietary fibre and for health and prevention against serious disease. Changing food choice behaviour by swapping from refined to whole grain will require a range of appealing products, clear labelling and significant promotion to change dietary patterns. This is particularly true for countries where refined grain intake is the norm and white bread and white rice are staple products. For this transition to whole grain to be successfully achieved, carefully constructed sensory studies that take into account commercialisation opportunities are required. 

## Figures and Tables

**Table 1:** Red and white wheat sensory studies published 1990–2019.

Author	Subjects	Study Title	Food Vehicle	Objectives	Key Results
McGuire and O’Palka [22]	*n* = 11 (but not all participants tested each sample over the 2 year period)	Sensory evaluation of a hard white compared to a hard red winter wheat	Bread (including wholemeal)	To determine differences in flavour or texture of bread made of wholemeal and milled flour from red and white wheat.	Colour differences were easily detected between red and white wheat and crust colour differences were significantly greater than crumb differences (*p = 0.018*). Panellists commented that red wheat bread had a harder, darker, bitter crust, whereas white wheat was more tender and sweeter.
Zhang and Moore [23]	*n* = 15	Wheat bran particle size effects on bread baking performance and quality	Bread (with added bran at 50 g, 100 g and 150 g kg^−1^)	To evaluate the effect of wheat bran particle size on bread baking performance and bread sensory quality comparing red and white wheat.	Red wheat bran was detrimental to loaf specific volume compared to breads containing other wheat brans. Soft white and soft red wheat bran breads were rated significantly differently for crust colour *(p < 0.05),* appearance, crumb, grain and texture. Panellists preferred bread made from soft white wheat bran *(p < 0.005)* and scored crust colour flavour *(p < 0.05)* and mouth feel *(p < 0.05)* higher than bread with red wheat bran *(p < 0.05)*.
Camire, Bolton, Jordan, Kelley, Oberholtzer, Qiu, Dougherty [24]	*n* = 66 (68% female)	Colour influences consumer opinions of wheat muffins	Muffins (including whole grain)	To determine if colour of wheat (red versus white) muffins impacts consumer acceptability as well as ratings of perceived healthiness and assess how information of muffin composition influences consumer opinion.	No difference between muffins was found for the colour, appearance, flavour and texture prior to ingredients being revealed to the panellists. Once information was provided, coloured (refined flour) muffins acceptability decreased significantly *(p < 0.05)* compared to red whole wheat muffins. Overall, red whole wheat muffins received the highest score, followed by white whole wheat and the muffins from all-purpose flour received the lowest scores.
Challacombe, Seetharaman, and Duizer [8]	Trained panellists: *n* = 13 (bread) and 10 (crackers) Untrained panellists: *n* = 73 (bread) and 76 (crackers)	Sensory Characteristics and Consumer Acceptance of Bread and Cracker Products Made from Red or White Wheat	Bread and crackers (including bran)	To compare intermediate moisture (bread) and low moisture (cracker) products made with red and white wheat, including bran particle sizes of whole grain products and consumer preferences.	Consumer acceptance: The breads and crackers produced from red wheat were liked significantly more in terms of appearance, flavour, texture *(p < 0.05)* and overall taste. Within the bread crust, no differences in the sensory properties were found. White wheat crumb was perceived as more bitter *(p < 0.05)* and less sweet *(p < 0.05)* than the red wheat crumb. White wheat crackers were scored as hard *(p < 0.05)* and earthy *(p < 0.05)*.
Watts, Ryland, Malcolmson, Ambalamaatil, Adams and Lukow [33]	*n* = 10 panellists	Flavour properties of pan and pita breads made from red and white hard spring wheat	Pan and pita bread (including whole grain)	To compare flavour properties of pan and pita bread made from red and white hard spring Canadian wheat.	Milder flavour properties were found for both the pan and pita breads made from the white wheat cultivars. All red wheat breads were significantly darker than the white wheat products in visual colour assessment tests *(p < 0.05).*
Keeney, Gol Mohamadi, Tsao, Planck and Ramsay [25] (abstract only)	*n* = 26 children aged 3–5 years (number of parents not reported)	Identification of Preferences for Hard White Wheat, Hard Red Wheat and Non-Whole Grain Bread Products in Young Children and Their Parents	Bread (including whole grain)	To identify children’s preferences for bread made from white wheat, red wheat and non-whole grain wheat, and identify parent bread preferences and purchasing habits.	At baseline 92% of children preferred bread made from white wheat, but no significant difference was identified in hedonic testing. Children consumed significantly more of the white wheat bread (3.7 g) than red wheat bread (3.3 g). P value not reported.
Worden, Keeney, Smith, Tsao and Ramsay [26] (abstract only)	*n* = 63 children aged 3–5 years (number of parents not reported)	Taste Preferences of Whole Grain Bread and Tortilla Products in Young Children: A Comparison of Hard White Wheat Versus Hard Red Wheat	Whole grain bread and tortillas (including whole grain)	To assess children’s liking of whole grain bread and tortillas made from hard red wheat and hard white wheat, and assess parents’ purchasing and consumption patterns.	65% of parents/children reported consuming whole grain bread but only 10% of children and 18% of adults consumed whole grain tortillas. Children reported liking bread made from white wheat (*n* = 29) and white wheat that was dyed (*n* = 32) more than the red wheat bread (*n* = 27) or the red wheat that had been dyed (*n* = 28). The children disliked tortillas made from red wheat (*n* = 16). Overall, there was greater liking and higher consumption of bread and tortillas made from hard white wheat but this did not reach significance.

**Table 2:** Red and white wheat sensory with physico-chemical analysis published 1990–2019.

Author	Subjects	Study Title	Food Vehicle	Objectives	Key Results
Lang and Walker [1]	*n* = 83 (for whole wheat tests)	Hard white and Red Winter wheat comparison in Hamburger Buns	Hamburger buns (including whole grain)	To create a high fibre hamburger bun comparing cracked wheat, flaked wheat, bran and whole wheat from red and white wheat.	No significant difference in firmness between red and white wheat buns. The flakes, bran of white wheat flour gave the lightest coloured buns compared to red wheat. Sensory evaluation: Despite the significant difference in taste *(p ≤ 0.001),* panellists had no clear preference for either wheat type or formulation (33/83 preferred white; 30/83 preferred red and 20/83 gave equal preference).
Chang and Chambers [27]	*n* = 6 (trained panellists with >300 h experience)	Flavour Characterization of Breads Made from Hard Red Winter Wheat and Hard White Winter Wheat	White pan bread and whole wheat bread (including whole grain)	To establish flavour profiles of breads made from red wheat and white wheat, and compare their flavour differences.	Whole wheat breads: In red wheat crumb, a browned impression was part of the grain complex, whereas in white wheat crumb, a toasted impression was part of the grain complex. In the crust, more after-taste remained from white wheat crumb than from red wheat crumb. NOTE: Possible contamination of whole white wheat flour with machine oil.
Chang, Seitz and Chambers [28]	*n* = 4 (trained panellists with >500 h experience)	Volatile Flavour Components of Breads Made from Hard Red Winter Wheat and Hard White Winter Wheat	Bread (including whole grain)	To isolate and characterise volatile flavour components from white (refined) bread versus whole wheat made from red and white wheat, and to compare the volatile flavour differences between white wheat and hard red wheat.	Fifteen compounds were significantly higher in whole wheat bread compared to white refined flour bread (*p < 0.005*). Differences were noted in volatile compounds between crust and crumb. Few differences were detected between breads made with HWW and HRW whole wheat flours with four compounds identified as being higher in red wheat and two higher in white wheat (*p < 0.005*).
Ramirez-Wong, Walker, Ledesma-Osuna, Torres, Medina-Rodriguez, López-Ahumada, Salazar-Garcia, Ortega-Ramirez, Johnson, Flores [36]	*n* = 50	Effect of flour extraction rate on white and red winter wheat flour compositions and Tortilla texture	Tortilla (including whole grain at 74%, 80% and 100% extraction)	To evaluate the physical, chemical and rheological properties of commercial flours made from white and red pericarp hard wheats milled to different extraction rates, and rate acceptability and storage stability of white and red wheat tortillas.	Chemical and physical evaluation: Dry gluten content was higher for white wheat (11.1%) than for red wheat (10.8%). Tortilla physical properties: white wheat tortillas had higher moisture contents than red wheat tortillas. White wheat had best colour at 80% extraction. White wheat had higher extraction rates and there was little change in tortilla firmness and colour. Results of sensory analysis showed that tortillas from both wheat flours and different extraction rates were well accepted.
Challacombe, Abdel-Aal, Seetharamana, Duizer [34]	*n* = 13 (bread) and 10 (crackers)	Influence of phenolic acid content on sensory perception of bread and crackers made from red or white wheat	Bread and crackers (including whole grain)	To quantify the relationship between total phenolic acid content and phenolic acids present within whole grain red and white wheat products. To determine if a relationship existed between the sensory properties of intermediate moisture (bread) products and low moisture (cracker) products made with flours from red and white wheat flours.	Despite having similar total phenolic acid content, red and white wheat products have different phenolic acid profiles. Soft red wheat flour contained more ferulic acid equivalents (1489.3 µg) than white wheat flour (1349.9 µg) *(p < 0.05).* Bound phenolics were significantly higher in products made with white wheat flour in comparison to those made with red wheat flour. The opposite trend for free phenolics was observed in red wheat bread. For the cracker products, free phenolics were higher in red wheat (mostly Ferulic acid). Overall, temperature and moisture content appear to affect bound and free phenolics as well their degradation to simpler phenolics. Sensory results: Overall, red and white wheat bread and crackers had stronger grain-like flavours when compared to the control. Strong correlation between total phenolic acid concentration and whole grain sensory attributes in crumb and crackers.
Talbert, Hofer, Nash, Martin, Lanning, Sherman and Giroux [29]	*n* = 24 panellists	Hard White Versus Hard Red Wheat: Taste Tests and Milling and Baking Properties	Whole wheat bread and noodles (including whole grain)	To compare hard red spring wheat cultivars that had been converted to near-isogenic white-seeded versions, and test high and low polyphenol oxidase (PPO) levels.	Whole wheat bread made from white-seeded hard wheat was judged to have a sweeter taste (*p < 0.05*). PPO activity did not impact most flour, bread or noodle quality traits, but white wheat with low PPO for noodles was recommended and deemed advantageous for Asian noodles and wholemeal bread.

**Table 3:** Red and white wheat physico-chemical studies published 1990–2019.

Author	Study Title	Food Vehicle	Objectives	Key Results
Park, Shelton, Peterson, Martin, Kachman and Wehling [30]	Variation in Polyphenol Oxidase Activity and Quality Characteristics AmongHard White Wheat and Hard Red Winter Wheat Samples	Flour and grain (including whole grain)	To assess PPO activity in grain and flour of red and white wheat grown in different locations and based on genotype of the cultivars.	PPO activity among hard white wheat ranged from 267 to 1264 units/g for grain and 34 to 153 units/g for flour. PPO activity in hard red samples was 1268 units/g and 1015–1505 units/g for grain and 115 units/g with a range of 74–189 units/g for flour. Growing location and genotype had a significant effect on grain colour variation and flour PPO activity in hard white wheat samples, which may indicate selection strategies can be used. No such differences were found for hard red wheat samples.
Kim, Tsao, Yang and Cui [35]	Phenolic acid profiles and antioxidant activities of wheat bran extracts and the effect of hydrolysis conditions	Red and white wheat bran	To qualify and quantify phenolic acids in wheat bran from red and white wheat, and investigate the effect of hydrolysis and extraction conditions on the yield and profile of phenolic acids in red and white wheat bran.	All fractions of the red wheat bran had higher total phenolic content than their corresponding white wheat bran contributing to antioxidant activity. The concentrations of individual phenolic acids in red bran were higher than those in white bran (including ferulic, vanillic and syringic).
Ma, Li, Zhang, Wang, Qin, Ding, Xie and Guo [37]	Accumulation of Phenolic Compounds and Expression Profiles of Phenolic Acid Biosynthesis-Related Genes in Developing Grains of White, Purple, and Red Wheat	Grains of purple, red and white wheat	To investigate the expression profiles of phenolic acid biosynthesis in genes of developing grains of white, red and purple wheat, and provide a better understanding of phenolic acid biosynthesis in red, white and purple wheat grains.	Despite similar TPC content of the three wheat varieties, the bound ferulic acid and vanillic acid content of purple wheat varieties were significantly higher than those of white and red wheat. White wheat had significantly lower bound p-coumaric acid content. Soluble ferulic acid and vanillic acid were higher in purple and red wheat than white wheat. Low levels of syringic acid were detected in white wheat cultivars. White wheat and red wheat had the highest phenolic acid contents during early seed development, while the levels of phenolic acids during later development were highest in purple wheat.

Glossary of terms: AACC: American Association of Cereal Chemists; AHPA: acid-hydrolysable phenolic; HRW: hard red wheat; HWW: hard white wheat; L*: lightness is a term used in colourimetry to identify the colour properties of food and other items; PLS regression: partial least square regression; PPO: polyphenol oxidase is an enzyme which catalyses the oxidation of phenols in reactive species; PCR: polymerase chain reaction; TPAC: total phenolic acid content; TPC: total phenolic content.
